# Ectopic Expression of Pumpkin NAC Transcription Factor CmNAC1 Improves Multiple Abiotic Stress Tolerance in *Arabidopsis*

**DOI:** 10.3389/fpls.2017.02052

**Published:** 2017-11-28

**Authors:** Haishun Cao, Li Wang, Muhammad A. Nawaz, Mengliang Niu, Jingyu Sun, Junjun Xie, Qiusheng Kong, Yuan Huang, Fei Cheng, Zhilong Bie

**Affiliations:** Key Laboratory of Horticultural Plant Biology, Ministry of Education, College of Horticulture and Forestry Sciences, Huazhong Agricultural University, Wuhan, China

**Keywords:** pumpkin, ABA, NAC domain protein, ABRE, abiotic stress tolerance

## Abstract

Drought, cold and salinity are the major environmental stresses that limit agricultural productivity. NAC transcription factors regulate the stress response in plants. Pumpkin (*Cucurbita moschata*) is an important cucurbit vegetable crop and it has strong resistance to abiotic stress; however, the biological functions of stress-related NAC genes in this crop are largely unknown. This study reports the function of CmNAC1, a stress-responsive pumpkin NAC domain protein. The CmNAC1-GFP fusion protein was transiently expressed in tobacco leaves for subcellular localization analysis, and we found that CmNAC1 is localized in the nucleus. Transactivation assay in yeast cells revealed that CmNAC1 functions as a transcription activator, and its transactivation domain is located in the C-terminus. *CmNAC1* was ubiquitously expressed in different organs, and its transcript was induced by salinity, cold, dehydration, H_2_O_2_, and abscisic acid (ABA) treatment. Furthermore, the ectopic expression (EE) of *CmNAC1* in *Arabidopsis* led to ABA hypersensitivity and enhanced tolerance to salinity, drought and cold stress. In addition, five ABA-responsive elements were enriched in *CmNAC1* promoter. The *CmNAC1*-EE plants exhibited different root architecture, leaf morphology, and significantly high concentration of ABA compared with WT *Arabidopsis* under normal conditions. Our results indicated that CmNAC1 is a critical factor in ABA signaling pathways and it can be utilized in transgenic breeding to improve the abiotic stress tolerance of crops.

## Introduction

Crops are affected by all kinds of abiotic stresses such as drought, cold, heat, and salinity stress that could lead toward extensive production losses worldwide. Therefore, understanding the stress response and improving the stress tolerance of crops is critical to boost agricultural productivity, ensure food security, and environmental sustainability (Mittler, 2006; Zhu, 2016). As a largest plant-specific transcription factor family, NAC domain proteins play an important role in plant development and regulation of abiotic stress tolerance. These proteins recently have received the attention as a major regulator in various stress signaling pathways and have been found to improve the abiotic stress tolerance of different crops through genetic engineering (Hu et al., 2006; Nakashima et al., 2007; Hong et al., 2016; Huang L. et al., 2016; Wang et al., 2017). As transcriptional factors, NAC domain proteins contain highly conserved DNA-binding domain in the N-terminal and diverse transcription activation or repression domain in the C-terminal (Olsen et al., 2005; Kim et al., 2007; Hao et al., 2010).

The NAC domain transcription factors in *Arabidopsis* function in plant development, senescence, and stress regulation. *NAC1* and *AtNAC2* regulate root development (Xie et al., 2000; He et al., 2005); *CUC1*, *CUC2*, and *CUC3* control leaf serration and axillary bud development (Nikovics et al., 2006; Raman et al., 2008); *SND1* and *VND7* trigger the *de novo* xylem formation and regulate secondary wall synthesis in fibers (Zhong et al., 2006; Reusche et al., 2012). According to some recent reports, NAC domain proteins are also associated with senescence, such as *ORE1*, *NAP*, *ATAF1*, and *JUNGBRUNNEN1* play an important role in promoting or delaying senescence (Balazadeh et al., 2010; Wu et al., 2012; Yang et al., 2014; Garapati et al., 2015; Qiu et al., 2015; Takasaki et al., 2015). NAC domain proteins also act as major regulators in abiotic stress response, for instance *ATAF1*, *RD26*, *ANAC096*, and *ANAC013* are involved in the regulation of salinity, oxidative, and drought stress tolerance (Fujita et al., 2004; Wu et al., 2009; Clercq et al., 2013; Xu et al., 2013). In addition, NAC domain proteins have become a popular research topic for different plant species. The expression of *OsNAC6* and *ONAC022* genes is upregulated by various stresses, whereas transgenic rice plants enhance multiple stress tolerance (Nakashima et al., 2007; Hong et al., 2016). The overexpression of stress-responsive NAC domain protein, SNAC1, significantly improves the drought tolerance in the field while showing no yield penalty (Hu et al., 2006). *GmNAC11* and *GmNAC20* from soybean are induced by phytohormones and abiotic stress with different transcriptional activities. GmNAC11 is a transcriptional activator, whereas GmNAC20 is a mild transcriptional repressor. The EE of *GmNAC20* improves the salt and freezing tolerance and promotes the lateral root formation in *Arabidopsis*. By contrast, the EE of *GmNAC11* in *Arabidopsis* plants enhances only the salt tolerance (Hao et al., 2011). These findings revealed that NAC domain proteins are important regulators of plant development and stress response.

Cucurbits are one of the most important fruit vegetables cultivated worldwide. Most cucurbits, particularly watermelon, cucumber, and melon, are sensitive to abiotic stresses, such as salinity, cold, and drought (Xie et al., 2015). The use of stress tolerant pumpkin as rootstock was recently found to improve the salt tolerance of cucumbers and the low temperature, low magnesium and nitrogen stress tolerance of watermelons (Huang et al., 2010; Huang Y. et al., 2016; Xu et al., 2016; Nawaz et al., 2016, 2017; Niu et al., 2017). However, the possible molecular regulatory mechanism underlying pumpkin response to abiotic stress are not yet elucidated. Thus, an abiotic stress-related NAC transcription factor, *CmNAC1*, was characterized from the transcriptome data of salt-treated pumpkin. Expression patterns of *CmNAC1* in response to dehydration, salinity, cold, ABA, GA, and H_2_O_2_ treatments were analyzed through real-time quantitative PCR (RT-qPCR). To evaluate its function in abiotic stress tolerance, we expressed *CmNAC1* in *Arabidopsis* ectopically. Morphological assays revealed that the EE of *CmNAC1* altered the plant phenotypes, such as relative dwarfism and large roots under normal growth conditions. Moreover, *CmNAC1*-EE plants showed good tolerance under abiotic stress treatment. In summary, our results suggest that *CmNAC1* plays positive roles in plant stress response and can be a candidate gene for the improvement of crops stress tolerance in the future.

## Materials and Methods

### Plant Materials and Abiotic Stress Treatment

N15 an inbred line of pumpkin (*Cucurbita moschata Duch.*) developed at our laboratory was utilized as experimental material in this study. The seeds were germinated in distilled water and cultivated under a 12h light/12h dark cycle at 28°C/18°C in a growth chamber and photosynthetic photon flux density of 200 μmol m^-2^ s^-1^. The seedlings preparation method is described in our previous study (Xie et al., 2015). Fourteen-day-old pumpkin seedlings were treated with dehydration, 100 mM NaCl, 100 μM exogenous GA, 100 μM exogenous ABA, and 10 mM H_2_O_2_ at 4°C for 0, 0.5, 1, 4, 8, and 12 h. Different organs, including roots, young leaves, cotyledons, and hypocotyls (at the three-leaf stage); mature leaves and stems (at the six-leaf stage); and fruits (3 days after pollination) were used in measuring the organ-specific expression patterns of *CmNAC1*. All collected samples were immediately frozen in liquid nitrogen and stored at -80°C. *Arabidopsis thaliana* Columbia-0 plants cultured in substrate were used for the transgenic study of *CmNAC1*.

### RNA Isolation and RT-qPCR

Total RNA was extracted using Tranzol (TransGen Biotech, Inc., Beijing, China), and reverse transcription was performed using 2 μg of RNA using HiScript II One Step RT-PCR Kit (Vazyme, Piscataway, NJ, United States) according to the manufacturer’s instructions. RT-qPCR was performed on Applied Biosystems QuantStudio system using an ABI 7500 real-time PCR machine (Applied Biosystems, Foster City, CA, United States) on it default PCR program with a reaction mixture volume of 10 μl. The melting curve was recorded after 40 cycles to verify the primer specificity by heating from 65°C to 95°C. One microliter of RNase-free H_2_O was also included in each plate as a control template. *CmEF-1*α, *CmCAC* (Obrero et al., 2011), and *AtActin2* were used as the internal references. The 2^-ΔΔ*ct*^ method was using to calculate relative gene expression values (Livak and Schmittgen, 2001). The Ct value of two reference genes was the square root of Ct_CmEF-1α_ × Ct_CmCAC_. The primers are listed in Supplementary Table S2. All RT –qPCR reaction results were obtained from three independent replicates.

### Cloning and Analysis of *CmNAC1* and *CmNAC1* Promoter

The *CmNAC1* cDNA sequence was obtained from the transcriptome data of salt-treated pumpkin root in our laboratory. The NCBI accession number of the transcriptome is SRP066227. Primer pairs (Supplementary Table S2) were designed to amplify the coding sequence (CDS). The PCR products were cloned into pEASYT1-Blunt vector (TransGen Biotech, Inc., Beijing, China) and transfected into *Escherichia coli* Tran5αT1 competent cells (TransGen Biotech, Inc., Beijing, China). Finally, the target gene in positive clone strains was sequenced (Tsingke, Inc., Beijing, China). Multiple alignments of amino acid sequences of NAC domain proteins were performed using MEGA software (version 5.1) and DNAMAN (version 7). *CmNAC1* promoter sequence was cloned from pumpkin genomic DNA using GenomeWalker Universal Kits (Takara, Shiga, Japan). The 1069 bp upstream from the start codon (ATG) of *CmNAC1* was selected for further analysis. The online search tool PlantCARE was used to detect putative *cis*-acting regulatory elements^1^.

### Subcellular Localization and Transactivation Assay Analysis

The full-length CDS of *CmNAC1* was amplified by PCR using 2× High-Fidelity Master Mix (Tsingke, Inc., Beijing, China), and the fragments were inserted into the *Stu*I site of the pH7LIC5.0-N-eGFP vector by using ClonExpress II One Step Cloning Kits (Vazyme, Piscataway, NJ, United States) to generate *35S*::eGFP-CmNAC1 fusion protein under the control of the *Cauliflower mosaic virus (CaMV) 35S* promoter (Zhang et al., 2015). The construct and negative control (pH7LIC5.0-N-eGFP) were transformed into Agrobacterium strain GV3101 and were infiltrated into tobacco (*Nicotiana benthamiana*) leaves via *Agrobacterium*-mediated transformation method (Sheludko et al., 2007). Laser scanning confocal microscope was used to detect the GFP fluorescence signal. The nucleus was stained with DNA dye 4,6-diamidino-2-phenylindole (DAPI).

The CDS of *CmNAC1* and the sequence encoding the N-terminus (1–450 bp) and C-terminus (450–879) were cloned. The PCR products were amplified with primers listed in Supplementary Table S2 and inserted into the *BamH*I site of pGBDKT7 vector and fused with the DNA-binding domain by using ClonExpress II One Step Cloning Kits to obtain pGBDKT7-CmNAC1-FL (1–292 aa), pGBDKT7-CmNAC1-N (1–150 aa) and pGBDKT7-CmNAC1-C (150–292 aa) constructs. These vectors and the pGBDKT7 (negative control) were separately transformed into the yeast strain Y2HGold. The transformed yeast cells were incubated on SD/-Trp, SD/-Trp/-His-Ade and SD/-Trp-His-x-gal plates. The transactivation activity was detected according to their growth status and α-galactosidase activity.

### Generation of *CmNAC1* Transgenic *Arabidopsis* Plants

The CDS of *CmNAC1* was cloned into the pHellgate8 vector to generate the 35S::*CmNAC1* construct by ClonExpress II One Step Cloning Kits. The construct was transformed into *Agrobacterium tumefaciens* strain GV3101 and then transferred into *Arabidopsis thaliana* ecotype Col-0 plants using the floral dip method (Clough and Bent, 1998). Transgenic *Arabidopsis* seeds were screened on MS medium suspended with kanamycin (50 mg/L). T3 homozygous lines were used for further experiments.

### Abiotic Stress Tolerance Assays and ABA Sensitivity Analysis

The WT and *CmNAC1*-EE lines were used to evaluate the tolerance for various abiotic stresses. For the drought treatment, the water intake of 4-week-old potted *Arabidopsis* plants in water-saturated substrate was withheld for 2 weeks. Subsequently, the plants were re-watered for 7 days. Another batch of seedlings grown under normal conditions was subjected to -10°C cold treatment for 1 h to characterize the freezing tolerance of transgenic plants. These plants were incubated in 4°C growth chamber for 3 h before transferring to normal growth conditions (23 ± 1°C) for recovery. For the salt tolerance assay, 4-week-old potted *Arabidopsis* plants were subjected to 250 mM NaCl treatment for 2 weeks. The survival rate of transgenic and WT *Arabidopsis* lines were scored after each kind of stress treatment. The experiments were independently repeated three times and used approximately 40 plants each.

For ABA sensitivity analysis of transgenic plant, *Arabidopsis* seeds were cultivated on 1/2 MS medium supplemented with 0 and 2 μM ABA medium under 16 h light/8h dark cycle at 23°C/18°C in a growth chamber. The germination rate (seedlings with cotyledon) was scored from the 5th to 8th day after planting on the plates. The 7-day-old seedlings were transferred to vertical plates supplemented with 0 and 10 μM ABA for ABA response analysis. Images of roots and shoots were captured and scored using WinRHIZO Pro 2013 image analysis system.

### Measurement of Electrolytic Leakage, Chlorophyll, ABA Content, Water Loss Rate, and Chlorophyll Fluorescence

Imaging PAM (MAXI; Heinz Walz, Effeltrich, Germany) was used to measure the chlorophyll fluorescence *Fv/Fm* value after the seedlings were stored in the dark for 30 min according to a previous method (Cheng F. et al., 2016). Fully expanded leaves from the 5-week-old seedlings of WT and EE plants were excised and weighed immediately to calculate the water loss rate. The leaves were dehydrated for 3 h on the dry filter paper (24°C–26°C) and weighed at designated time points. Images were captured at 0 and 3 h after treatment. The water loss rate was calculated based on the initial fresh weight of the leaves. Before and after cold treatment, the 4-week-old seedling leaves were collected and sampled for the measurement of electrolytic leakage according to a previous method (Hong et al., 2003). The relative chlorophyll content of 6-week-old seedling leaves from the control and salt-treated plants were measured as SPAD value using chlorophyll meter SPAD-502. For ABA content measurement, 0.10 g of 5-week-old seedling leaves were sampled and grinded on ice with cold extraction buffer (methanol:water:acetic acid, 80:19:1, v:v:v), shaken for 16 h at 4°C, centrifuged at 13,000 rpm for 15 min at 4°C. The supernatant was supplemented with internal standards, filtered using a syringe-facilitated 13-mm diameter nylon filter, dried by evaporation, and dissolved in 200 μl methanol. Simultaneous quantification of ABA by liquid chromatography was conducted using a UFLC with an autosampler according the method (Pan et al., 2008; Liu et al., 2012).

The experiment was repeated three times at least. All data were compared using Student’s *t*-test for statistical analysis.

## Results

### *CmNAC1* Is a Stress-Responsive Gene

**FIGURE 1:**
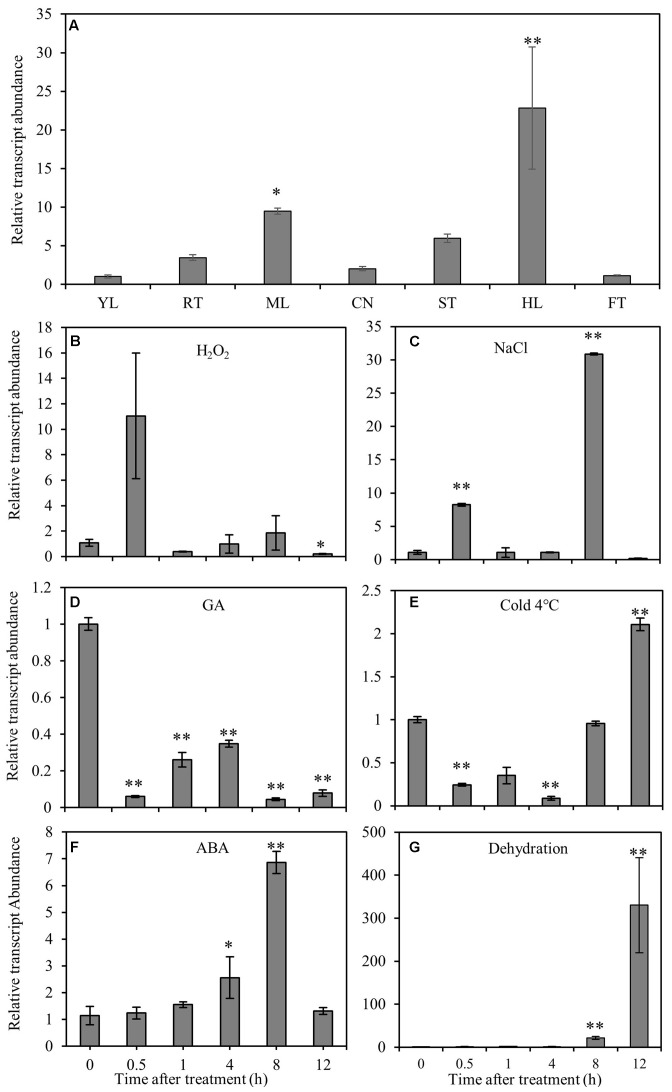
Expression patterns of *CmNAC1* in pumpkin seedlings under different stress treatments or in different organs. **(A)** Expression patterns of *CmNAC1* in various organs (root, RT; young leaves, YL; mature leaves, ML; cotyledon, CN; hypocotyl, HL; stem, ST; fruit, FT). Expression patterns of *CmNAC1* in leaves after H_2_O_2_ **(B)**, NaCl **(C)**, GA **(D)**, cold **(E)**, ABA **(F)** and dehydration **(G)** treatments by qRT-PCR analysis. Zero represents leaf sample without any treatment; 0.5, 1, 4, 8, and 12 represent samples after 0.5, 1, 4, 8, and 12 h treatment, respectively. The 2^-ΔΔCT^ method was used in qRT-PCR analysis. Transcript levels were normalized to *CmEF1*α and *CmCAC*. Values are means ± SD of three replicates. Three independent experiments were performed. Asterisks indicate a significant difference (^∗^*P* < 0.05; ^∗∗^*P* < 0.01) compared with the corresponding controls.

RT-qPCR was performed to elucidate the gene expression pattern in different tissues and the response of *CmNAC1* to various abiotic stresses. Temporal and spatial expression results revealed that *CmNAC1* has relative high expression levels in mature leaves, stem, roots, and the highest in hypocotyl. However, its expression was low in the new leaves and fruits (**Figure 1A**). Under H_2_O_2_ and NaCl stress conditions, *CmNAC1* transcript was upregulated specifically at 0.5 and 8 h (**Figures 1B,C**). The expression of *CmNAC1* was induced during 0.5–8 h under dehydration and ABA treatment, but only the transcript levels in ABA treatment reverted at 12 h (**Figures 1F,G**). However, GA consistently inhibited *CmNAC1* expression during the treatment (**Figure 1D**). The cold stress repressed the *CmNAC1* expression within 4 h, and then the transcript levels were induced until 12 h (**Figure 1E**). The qRT-PCR results suggest that similar to other stress-associated NAC domain proteins, *CmNAC1* participates in plant stress response, and its expression is sensitive to ABA, H_2_O_2_, and GA signaling molecules.

The 1 kb upstream of ATG start codon *CmNAC1* promoter sequence was cloned to detect the putative, stress-responsive *cis*-acting regulatory elements (**Supplementary Figure S5**). Stress response-related *cis*-acting elements, that include five ABA-responsive elements (ABREs), one low-temperature responsive motif, two GC motifs involved in anoxic specific inducibility, and two TGACG motifs involved in methyl jasmonate responsiveness (**Table 1**), were found in the promoter. These stress-related *cis*-acting elements could be necessary for the stress-regulated expression of *CmNAC1*. These findings indicate that CmNAC1 plays a critical role in early stress responsiveness.

**Table 1:** Stress-related *cis*-acting regulatory elements identified in the promoter region of *CmNAC1.*

Site name	Position	Strand	Sequence	Function
ARE	120,1391	-	TGGTTT	*Cis*-acting regulatory element essential for anaerobic induction
TC-rich repeats	927	+	ATTTTCTCCA	*Cis*-acting element involved in defense and stress responsiveness
HSE	447	+	AAAAAATTTC	*Cis*-acting element involved in heat stress responsiveness
MBS	591, 1592	+	CAACTG	MYB binding site involved in drought-inducibility
MBS	158	-	CGGTCA	MYB binding site
LTR	860,1052	+	CCGAAA	*Cis*-acting element involved in low-temperature responsiveness
Crepeat/DRE	329	+	TGGCCGAC	Regulatory element involved in cold- and dehydration-responsiveness
ABRE	118,152 379,520	-	CACGTG	*Cis*-acting element involved in the abscisic acid responsiveness
ABRE	496	+	ACGTGGC	*Cis*-acting element involved in the abscisic acid responsiveness
TCA-element	1095	+	TCAGAAGAGG	*Cis*-acting element involved in salicylic acid responsiveness
TCA-element	750	-	CCATCTTTTT	*Cis*-acting element involved in salicylic acid responsiveness
TGACG-motif	362,1428	+	TGACG	*Cis*-acting regulatory element involved in the MeJA-responsiveness
CGTCA-motif	1775	-	CGTCA	*Cis*-acting regulatory element involved in the MeJA-responsiveness
TATCCAT/C-motif	1894	-	TATCCAT	*Cis*-acting regulatory element; associated with G-box like motif; involved in sugar repression responsiveness
W box	1514, 1344	-	TTGACC	Elicitation; wounding and pathogen responsiveness. Binds WRKY type transcription factors
W box	631, 783	+	TTGACC	Elicitation; wounding and pathogen responsiveness. Binds WRKY type transcription factors


### CmNAC1 Belongs to ATAF Subfamily with Transcriptional Activity and Is Localized in the Nucleus

*CmNAC1* cDNA had a length of 1200 bp and was predicted to contain an 879 bp CDS encoding 292 amino acid protein. The *CmNAC1* gene accession number in NCBI GenBank database was MG199592. Amino acid sequence alignment shows that CmNAC1 shared an identity of 65.35% with AtATAF1 protein from Arabidopsis. CmNAC1 protein also contained a diverse activation domain in C-terminal and a conserved NAC domain in the N-terminal region including subdomains A to E (**Supplementary Figure S1**). To further reveal the divergence of CmNAC1 protein during evolution, we analyzed the phylogenetic relationship of CmNAC1 with SNACs orthologues, which were functionally characterized in *Arabidopsis* and crops. Phylogenetic analysis showed that *CmNAC1* and *AtATAF1* gene are in the same clade (**Supplementary Figures S2**, **S7**).

**FIGURE 2:**
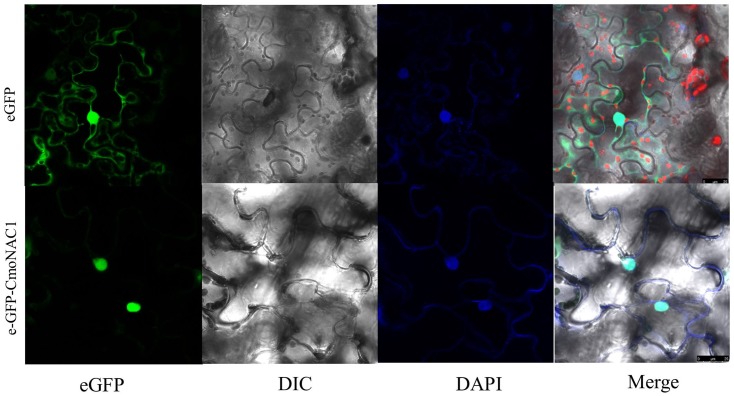
Nuclear localization of CmNAC1. e-GFP and e-GFP-CmNAC1 fusion proteins were transiently expressed in tobacco leaves under control of the *CaMV 35S* promoter and observed under a laser scanning confocal microscope. e-GFP images, DAPI stained images, differential interference contrast images (DIC), and merged images were taken.

The eGFP-CmNAC1 fusion construct and the eGFP control in pH7LIC5.0-N-eGFP vector driven by *CaMV35S* promoter were transiently expressed in tobacco epidermal cells and visualized under a laser scanning confocal microscope to determine the subcellular localization of CmNAC1. The eGFP fluorescence signal was widely observed from the cytoplasm to nucleus, whereas the eGFP-CmNAC1 fusion protein fluorescence signal was mainly detected in the nucleus as confirmed by DAPI staining (**Figure 2**). Thus, above results demonstrated that CmNAC1 is a nuclear protein.

**FIGURE 3:**
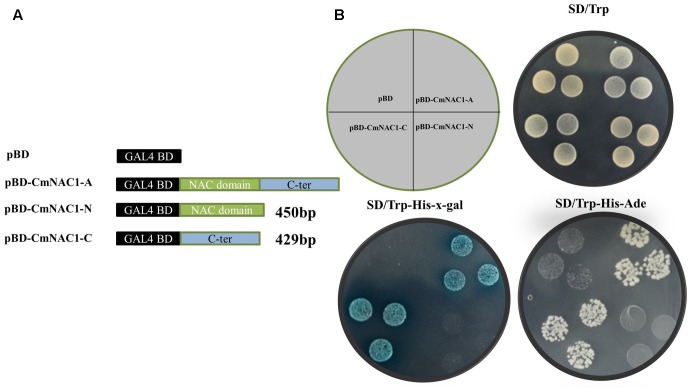
Transactivation assay of CmNAC1 in yeast cell Y2HGold. **(A)** Full-length protein (CmNAC1-A), N-terminal fragment (CmNAC1-N) and C-terminal fragment (CmNAC1-C) were fused with GAL4 DNA binding domain and expressed in yeast strain Y2HGold. The pGBDKT7 vector was used as a negative control. **(B)** Transformed yeasts were dripped on the SD/-Trp, SD/-Trp-His-Ade, and SD/-Trp-His-x-gal after being cultured for 3 days in the growth chamber.

The entire coding region, N-terminal and C-terminal domain coding sequence were inserted into the pGBDKT7 vector, which contains the GAL4 DNA-binding domain, to investigate the transcriptional activity of CmNAC1 protein (**Figure 3A**). These constructs and empty vector pGBDKT7 (negative control) were transformed into the yeast strain Y2HGold. The transactivation results show that all transformed yeast cells grew well on SD/-Trp medium. The yeast strain containing the full-length CmNAC1 (pGBDKT7-CmNAC1-A) and the C-terminus of CmNAC1 (pGBDKT7-CmNAC1-C) could grow well on the selected medium SD/-Trp/-His/-Ade, whereas the cells with the N-terminus of CmNAC1 (pGBDKT7-CmNAC1-N) and pGBDKT7 empty vector could not grow normally. Furthermore, the yeast cells that grew well on the SD/-Trp/-His-x-gal medium appeared blue in the presence of α-galactosidase, indicating the activation of the reporter gene LacZ (**Figure 3B**). These results indicate that CmNAC1 is a transcriptional activator, and its transactivation domain localizes in the C-terminus.

### Ectopic Expression of *CmNAC1* Changes the Phenotype of *Arabidopsis*

**FIGURE 4:**
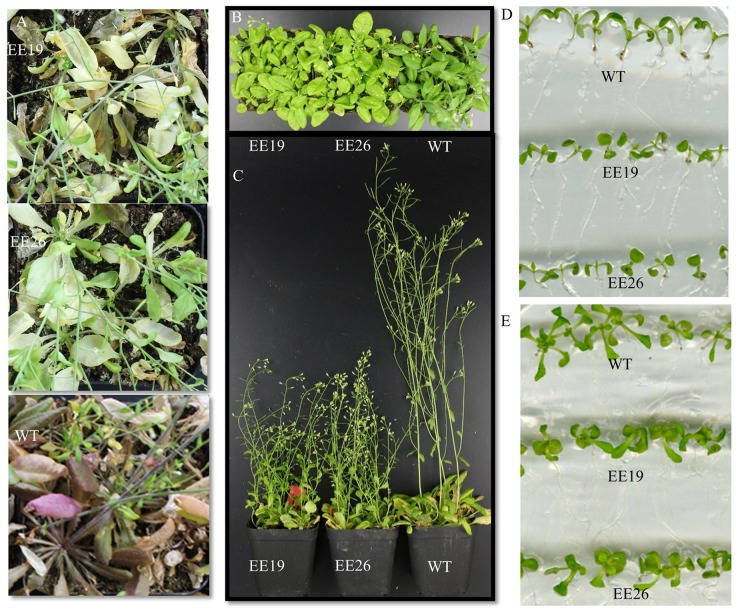
Phenotypic analysis of *CmNAC1*-EE lines. **(A)** Seven-week-old WT and *CmNAC1*-EE plants were grown in substrate under long-day (LD) conditions. **(B)** Five-week old WT and *CmNAC1*-EE plants grown in substrate under LD conditions. The leaves of *CmNAC1*-EE lines were light green. **(C)** The plant height of WT and *CmNAC1*-EE plants grown under LD conditions after 6 weeks. **(D)** Transgenic plants grown in the 1/2 MS medium for 8 days. **(E)** Transgenic plants grown in the 1/2 MS medium for 16 days. EE lines show curly cotyledons and leaves.

**FIGURE 5:**
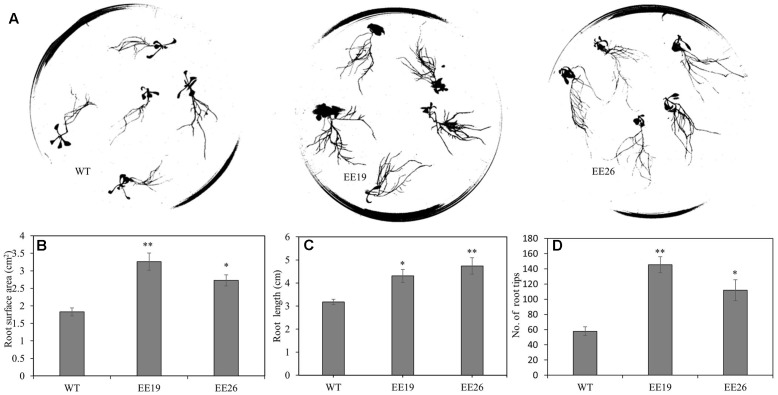
*CmNAC1*-EE lines have enhanced root growth. **(A)** Root scanning picture of WT, EE19 and EE26 lines. **(B)** Quantification of total root surface area. **(C)** Quantification of total root length. **(D)** Quantification of total root tips. Three independent experiments were performed. Asterisks indicate statistically significant differences compared with WT (^∗^*P* < 0.05; ^∗∗^*P* < 0.01).

When grown in the substrate, the *CmNAC1-*EE plants showed two different phenotypes. One was similar to WT, and the other was dwarf and light green in color (**Supplementary Figure S3B**). Eight transgenic positive lines with sufficient seeds and stable phenotype were selected for further gene expression experiment. RT-PCR results show that three EE lines had the same phenotype that is different from that of WT, and PCR analysis using vector specific primer indicated that all transgenic lines are positive (**Supplementary Figure S3A**). Therefore, *CmNAC1-*EE lines EE19 and EE26 with high *CmNAC1* expression levels were selected for further analysis. EE19 and EE26 lines showed sterility and dwarfism phenotype with light green and round-shaped leaves (**Figures 4B,C**). These plants also showed accelerated chlorophyll degradation during senescence (shown as white color) as compared with wild type plants with red senescence leaves (**Figure 4A**). When grown in the 1/2 MS dish, both EE line seedlings showed downward cotyledon and light color (**Figures 4D,E**). Also, both EE lines had larger roots with larger total root surface area, longer total root length, and more root tips than the WT plants (**Figure 5**). These findings suggest that *CmNAC1* is an important regulator of plant phenotype development.

### Ectopic Expression of *CmNAC1* Improves Drought Tolerance and Increases ABA Content in *Arabidopsis*

**FIGURE 6:**
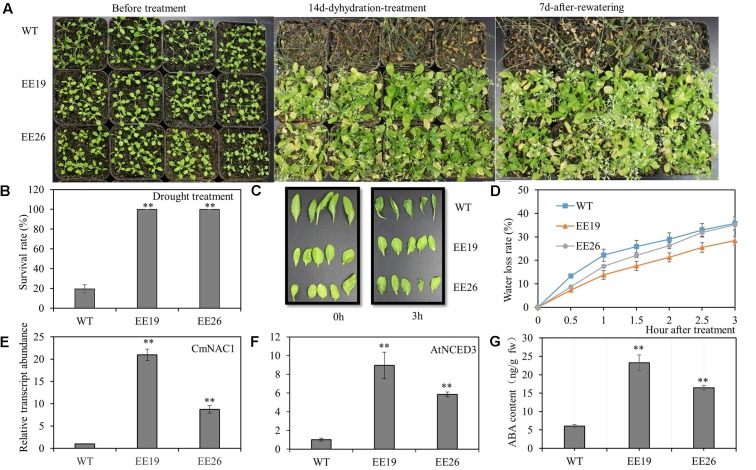
*CmNAC1*-EE lines have enhanced drought stress tolerance. Four-week-old seedlings of WT and EE plants were grown in substrate for treatment. **(A)** Phenotypes of WT and EE plants during drought stress. **(B)** Quantitative analysis of survival rate after drought stress. **(C)** Fully expanded leaves of *Arabidopsis* plants (5 weeks old) were excised and exposed to dehydration stress for 3 h on dry filter paper at room temperature. Pictures were taken at 0 and 3 h after treatment. **(D)** Water loss assay from detached leaves of 5-week-old seedlings. **(E)** *CmNAC1* expression levels in transgenic *Arabidopsis* EE19, EE26 lines and WT plants. **(F)** Key ABA biosynthesis gene *AtNCED3* expression levels in transgenic *Arabidopsis* EE19, EE26 lines and WT plants. **(G)** Concentration of ABA in transgenic *Arabidopsis* EE19, EE26 lines and WT plants. Three independent experiments were performed. Asterisks indicate statistically significant differences compared with WT plants (^∗∗^*P* < 0.01).

The 4-week-old transgenic lines EE19, EE26 and WT *Arabidopsis* seedlings were transferred to water-saturated substrate to determine the function of *CmNAC1* to drought stress tolerance in plants. Water was suspended to gradually reduce water availability in the substrate. After 2 weeks of treatment, most WT plants wilted because of the extreme water deficit. By contrast, the *CmNAC1*-EE *Arabidopsis* plants showed little wilting and a slight leaf senescence phenotype. After re-watering, most of the *CmNAC1*-EE plants remained vigorous and survived, whereas only few of the WT plants recovered (**Figure 6A**). Furthermore, the survival ratio of the two *CmNAC1* transgenic lines (100%) was higher than those of the WT plants (19.5%) under drought stress (**Figure 6B**). The *CmNAC1-* EE plants showed lower water loss rate and better water retaining phenotype than WT during the 3 h dehydration stress (**Figures 6C,D**). The EE plants also had significantly high expression levels of key ABA biosynthetic gene *AtNCED3* in accordance to *CmNAC1* expression levels and ABA content (**Figures 6E,F,G**). These results suggest that *CmNAC1*-EE leads to high ABA content and efficient water use in *Arabidopsis*.

### *CmNAC1* Ectopic Expressing Plants Are Tolerant to Salt and Cold stress

**FIGURE 7:**
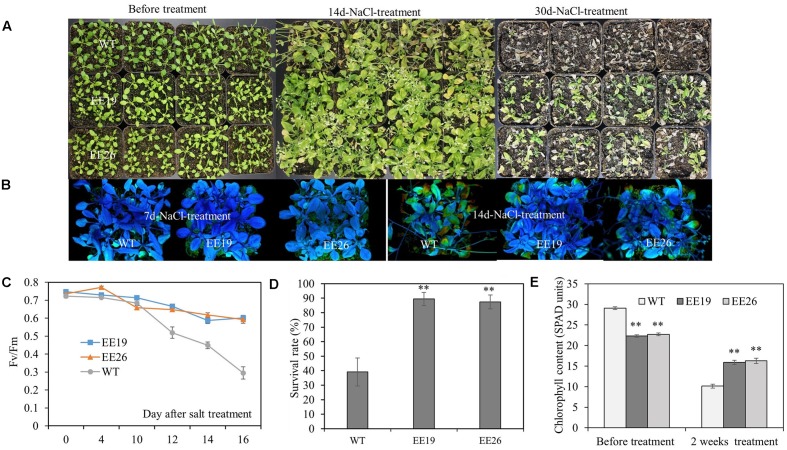
*CmNAC1*-EE lines have enhanced salt stress tolerance. Four-week-old seedlings of WT and EE plants were grown in substrate for NaCl (250 mM) treatment. **(A)** Phenotypes of WT and EE plants before salt stress and after 14 and 30 days of salt treatment. **(B)** *Fv/Fm* images during seven and 14 days after salt treatment. **(C)** *Fv/Fm* value was measured 0, 4, 10, 12, 14, and 16 days after salt treatment. **(D)** Survival rate after salt stress. **(E)** Chlorophyll content of *CmNAC1*-EE lines and WT plants was measured by SPAD before and after 14 days of salt treatment. Three independent experiments were performed. Asterisks indicate statistically significant differences compared with WT plants (^∗^*P* < 0.05; ^∗∗^*P* < 0.01).

Salt tolerance of *CmNAC1*-EE plants were examined. Four-week-old seedlings cultivated in substrate were irrigated with 250 mM NaCl solution. After 30 days of salt treatment, *CmNAC1*-EE lines showed better growth status than the WT plants, thereby indicating serious chlorosis (**Figure 7A**). As shown in **Figure 7C**, the *Fv/Fm* value decreased significantly starting at the 10th day of treatment, this indicated that the leaves were damaged starting at the 10th day of salt treatment, and the EE lines consistently performed better than WT *Arabidopsis*. Moreover, the Fv/Fm images also revealed that salt damage at the 7th day of treatment was not visible. However, at the 14th day of treatment, the WT plants were significantly damaged compared with the EE plants (**Figure 7B**). Consistent with the *Fv/Fm* value changes, the transgenic plants had lighter green leaves (lower SPAD value) without salinity. However, after 2 weeks of salt stress treatment, their SPAD value was higher than that of the WT plants (**Figure 7E**). All EE lines also had high survival rate (**Figure 7D**). In conclusion, EE lines are more tolerant to salinity stress.

Four-week-old potted *Arabidopsis* seedlings were subjected to freezing at -10°C for 1 h to characterize the function of *CmNAC1* in cold tolerance. After that, incubate the plants in a cold growth chamber (4°C) for 3 h, followed by transferring the plants to normal growth conditions (23 ± 1°C) for recovery. EE lines had less cold injury and better recovery than the WT plants after cold treatment (**Supplementary Figure S4A**). Moreover, the survival ratio of the two transgenic lines were significantly higher than WT plants (**Supplementary Figure S4B**). During cold treatment, the two *CmNAC1* transgenic lines also had lower electrolyte leakage than WT plants (**Supplementary Figure S4C**). These results indicate that EE of *CmNAC1* also improves the tolerance of plants to low-temperature stress.

### *CmNAC1* Ectopic Expressing *Arabidopsis* Lines Are ABA Hypersensitive

**FIGURE 8:**
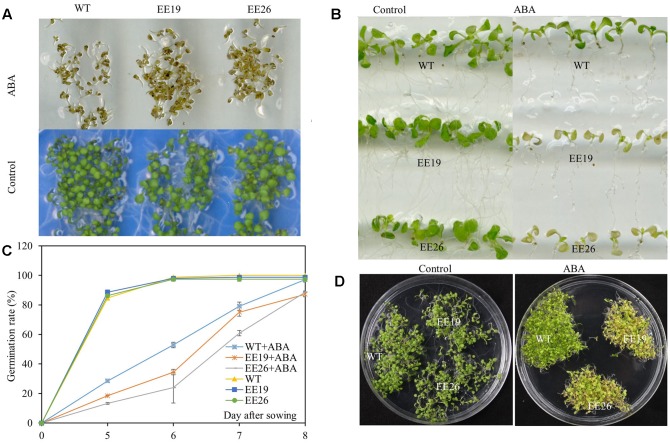
Hypersensitivity of *CmNAC1-*EE lines to ABA. Phenotypes of WT and EE plants grown in 1/2 MS medium supplemented with ABA for sensitivity quantitation. **(A)** Pictures of germination phenotype with 0 and 2 μM ABA were taken after 8 days of treatment. **(B)** Phenotype of WT and EE lines in vertical 1/2 MS medium with 0 and 10 μM ABA. **(C)** Germination rate were scored at five, six, seven, and 8 days after planting in the 1/2 MS plates supplemented with 0 and 2 μM ABA. **(D)** Phenotype of WT and EE lines grown in 1/2 MS medium with 0 and 2 μM ABA for 2 weeks and EE lines showing yellow phenotype compared with WT green phenotype.

Abscisic acid is a critical stress regulator in plants, and EE lines had high ABA content. Therefore, the ABA sensitivity of EE plants was assessed. ABA significantly inhibited *Arabidopsis* germination when the seeds were cultivated on 1/2 MS medium supplemented with 0 and 2 μM ABA (**Figure 8A**). However, the emergence ratio of WT seeds was higher than that of EE lines seeds in 1/2 MS medium containing 2 μM ABA (**Figure 8C**). The seedlings of 7-day-old WT and EE lines were transplanted to vertical 1/2 MS medium containing 0 and 10 μM ABA for ABA sensitivity analysis. Both EE lines showed yellow and senescent shoot phenotypes compared with the control (**Figure 8B**). Additionally, when the seedlings were cultivated in 1/2 MS medium supplemented with 2 μM ABA for 2 weeks, both transgenic lines showed yellow seedling cotyledon leaves compared with the light green seedling cotyledon leaves of WT plants (**Figure 8D**). Therefore, we suggested that *CmNAC1* transgenic line shoots are more hypersensitive to ABA than WT plants.

## Discussion

### CmNAC1 Is a Nucleus Localized Stress-Responsive NAC Transcriptional Activator

NAC proteins contain highly conserved DNA-binding domain; but, they play different roles in various stress signaling pathway and several developmental programs across different plants (Puranik et al., 2012). However, the function of NAC transcriptional factors in pumpkin is still unknown. In this study 76 NAC domain proteins were identified from the transcriptome data (**Supplementary Figure S6**), *CmNAC1* is the greatest induced NAC genes by salinity. CmNAC1 is also the first identified stress-related NAC transcription factor in pumpkin containing diverse C-terminal activation domain and conserved N-terminal NAC domain. Amino acid sequence alignment shows that CmNAC1 has high sequence identity with AtATAF1 of stress-responsive NAC domain proteins from *Arabidopsis* (**Supplementary Figure S1**). Recently plenty of NAC domain proteins were identified to be stress-responsive and those proteins have important function in different stress signaling pathways (Hao et al., 2011; Xu et al., 2013; Yang et al., 2015; Hong et al., 2016; Zhang et al., 2016). The expression of *CmNAC1* is induced by drought, salt, ABA and H_2_O_2_ (**Figures 1B,C,F,G**), and several stress-related *cis*-elements are present in promoter of *CmNAC1* (**Table 1**), suggesting it is a stress-responsive NAC domain protein. Most of transcription factors are localized in nucleus and have transactivation activity, CmNAC1 protein is also localized in nucleus (**Figure 2**) and both the C-terminal and full-length CmNAC1 have high transactivation ability in yeast (**Figure 3B**). However previous studies reported that the conserved hydrophobic LVFY motif in N-terminal of NARD would lead to abolished transactivation (Hao et al., 2010; Wang et al., 2017). CmNAC1 also contains NARD, but we have not observed significant difference of transactivation ability between the C-terminal and full-length CmNAC1. This might be due to its high transcriptional activation motif in C-terminal, but the mechanisms need to be further investigated. These results suggest that CmNAC1 is a nucleus localized stress-responsive transcriptional activator.

### *CmNAC1* Is Involved in Developmental Processes

Growth retardation with a dwarf phenotype has been observed in *Arabidopsis* EE of *NTM1*, *AtATAF1*, *OsNAC2*, *MlNAC5*, and *ANAC036* (Kim et al., 2006; Wu et al., 2009; Kato et al., 2010; Chen et al., 2015; Yang et al., 2015). *CmNAC1*-EE also leads to a dwarf phenotype (**Figure 4C**), indicating that *CmNAC1* regulates shoot development. Plants can change their phenotypes to alleviate the harmful impacts of environmental stresses (Pierik and Testerink, 2014). *AtATAF1* are reported to play an important role in promoting senescence and chlorophyll degradation (Garapati et al., 2015). *CmNAC1*-EE also showed light color and accelerated chlorophyll degradation during senescence with white leaves (**Figure 4A**). The results suggested the function of CmNAC1 are conserved in some aspects. Interestingly, we also found different root phenotype in EE lines. Several studies have proved that rice, *Arabidopsis*, and tobacco plants with optimized root systems have good tolerance to drought and limited nutrient supply (Yu et al., 2008; Werner et al., 2010; Uga et al., 2013). These findings are in agreement with our results that EE plants have large roots and good stress tolerance (**Figure 5**). *NAC1* and *AtNAC2* from *Arabidopsis* can promote later root development (Xie et al., 2000; He et al., 2005), and NAC domain proteins function as homo- or heterodimers (Olsen et al., 2005). However, the mechanisms of *CmNAC1* in regulating root development and its cooperation with other NAC transcriptional factors related with auxin and root architecture development are still unknown.

### *CmNAC1* Improves Tolerance to Multiple Abiotic Stress

Recently, numerous NAC domain proteins from different crops were reported to play a positive role in stress responsiveness and regulation of abiotic stress tolerance (Hong et al., 2016; Huang L. et al., 2016; Zhang et al., 2016; Wang et al., 2017). The observations that *CmNAC1* significantly improves the tolerance of *Arabidopsis* to salt, drought and cold stress and EE lines shows better growth performance and higher survival ratio under stress conditions (**Figures 6A,B**, **7A,D** and **Supplementary Figures S4A,B**) indicates that CmNAC1 also functions as a positive stress-responsive transcription factor of salt, drought and cold stress tolerance in pumpkin. In the represent study, several physiological changes in transgenic *Arabidopsis* seem involved in the mechanisms of abiotic stress tolerance. First, *CmNAC1* significantly improves the tolerance of *Arabidopsis* to salt stress, and reducing the accumulation of Na^+^ in leaves is one of plant salinity tolerance mechanisms (Roy et al., 2014). The leaves of *CmNAC1-*EE plants got less damaged when grown under salt stress (**Figures 7B,E**), it is might be due to less Na^+^ content in EE leaves. Secondly, as one of the most important phytohormones, ABA regulates plant development, closing of stomata, and stress tolerance (Raghavendra et al., 2010; Yoshida et al., 2014). It is observed that *CmNAC1-*EE *Arabidopsis* lead to higher ABA content and ABA hypersensitive (**Figures 6G**, **8**), this could lead to increased closing of stomata under drought, and then control water loss ratio and enhance drought tolerance (**Figures 6A,C,D**). Thus increased ABA content and sensitivity in EE plants may partially account for the enhanced drought tolerance, which is consistent with previous findings that overexpression of *MlNAC5*, *AtATAF1*, *ONAC022*, *TaNAC47* in plants led toward improved drought stress (Wu et al., 2009; Yang et al., 2015; Hong et al., 2016; Zhang et al., 2016). Finally, cold-responsive genes in plants were also partially regulated by ABA, ABA-mimicking ligand application could improve cold stress tolerance (Cheng Z.M. et al., 2016), and we also found that *CmNAC1* could improve cold tolerance (**Supplementary Figures S4A,B**). Our result are in accordance with the previous studies that ectopic expression of *MlNAC5*, *GmNAC20* and *TaNAC47* conferred ABA hypersensitivity and enhanced cold tolerance in *Arabidopsis* (Hao et al., 2011; Yang et al., 2015; Zhang et al., 2016), indicating that CmNAC1 may function as a positive regulator of abiotic stress tolerance in ABA-dependent pathway.

### *CmNAC1* Plays a Critical Role in ABA-Dependent Signaling

Stress induced ABA biosynthesis in leaves was mainly controlled by *AtNCED3* (Tan et al., 2003). AtATAF1 is a rapid stress response transcription factor that controls ABA biosynthesis by binding to *AtNCED3* promoters and enhancing its expression (Jensen et al., 2013; Garapati et al., 2015). *CmNAC1* has a close relationship with the *AtATAF1* in *Arabidopsis* (**Supplementary Figure S1**), *CmNAC1-*EE lines have high *AtNCED3* expression levels and ABA content (**Figures 6F,G**). Therefore we speculate that *CmNAC1* could bind to the promoter of *AtNCED3* to regulate its expression, and this might be a conserved pathway in regulating ABA synthesis in plants. In addition, the expression of *CmNAC1* is significantly induced by dehydration and ABA (**Figures 1F,G**). These results indicate that *CmNAC1* play dual role in ABA signaling responsiveness and ABA synthesis regulation. ABREs are recognized by a group of transcription factors, ABRE-binding factors (ABRE/ABFs) (Yoshida et al., 2014). In *Arabidopsis AREB1, AREB2*, and *ABF3* collectively regulate ABRE-dependent ABA signaling involved in drought stress tolerance (Yoshida et al., 2010). Several conserved ABREs were enriched in almost the exact position from promoter of *Arabidopsis AtATAF1*, cucumber *CsATAF1* (Supplementary Table S1), and rice *OsNAC6* (Nakashima et al., 2007). Similarly five ABREs are found in the promoter of *CmNAC1* (**Table 1**); suggesting that *ATAF1* homologous genes from different plant species have pivotal functions in ABRE-dependent ABA signaling. Therefore, *CmNAC1* might be a downstream gene of AREB/ABFs transcription factors and cooperatively regulate the ABRE-dependent gene expression. In summary, we can speculate that once the plants are exposed to stress, ABA quickly promotes *CmNAC1* expression through the AREB/ABF pathway. *CmNAC1* expression subsequently produces additional *NCED3* transcripts to finally obtain increased ABA in a positive feedback loop.

## Conclusion

The function of CmNAC1 as a new stress-response transcription factor in pumpkin was evaluated in *Arabidopsis* plants. *CmNAC1* plays a positive role in promoting root growth and adapting to salt, cold, and drought stress. The EE plants also had significantly high ABA level, indicating that the ABA signaling pathway is involved in *CmNAC1*-mediated abiotic stress tolerance. CmNAC1 is a functional transcription factor in pumpkin and a candidate gene for stress tolerance regulation and genetic modification of crops.

## Author Contributions

ZB, QK, YH, and FC designed the research. HC, LW, MLN, JS, and JX did experiment and analyzed the data. HC and MAN wrote the manuscript. The manuscript is revised by HC and ZB. All authors read and approved the manuscript.

## Conflict of Interest Statement

The authors declare that the research was conducted in the absence of any commercial or financial relationships that could be construed as a potential conflict of interest.

## Footnotes

1 http://bioinformatics.psb.ugent.be/webtools/plantcare/html/

## Supplementary Material

**FIGURE S1 |** Multiple alignment of CmNAC1 amino acid sequences and other NAC domain proteins from selected plant species. Alignment was performed using DNAMAN program. Four conserved NAC subdomains were indicated: GhNAC2 from *Gossypium hirsutum*, GmNAC2 from *Glycine max*, and CUC1 and AtATAF1 from *Arabidopsis thaliana*.

**FIGURE S2 |** Phylogenetic relationship of *CmNAC1* with *Arabidopsis* SNACs gene. *CmNAC1* from pumpkin and *ATAF1, ATAF2, RD26, ANAC102, ANAC055, SHYG, AtNAP, ORE1, ANAC056, ANAC016, ANAC018, ANAC019, ANAC025*, and *ANAC053* from *Arabidopsis thaliana*.

**FIGURE S3 |** Expression of *CmNAC1* in transgenic *Arabidopsis* lines. **(A)** PCR identification and RT-PCR results of *CmNAC1* expression levels in different transgenic lines. *AtActin* was used as the internal control gene. **(B)** Phenotypes of T0 generation plants grown for 5 weeks in substrate.

**FIGURE S4 |** Four-week-old seedlings of WT, EE19 and EE26 plants grown in substrate for cold treatment. **(A)** Phenotypes of EE lines and WT plants before and after cold stress treatment. **(B)** Survival rate quantitation. **(C)** Electrolyte leakage was measured during cold treatment. Asterisks indicate statistically significant differences compared with control (^∗^*P* < 0.05; ^∗∗^*P* < 0.01). Three independent experiments were performed.

**FIGURE S5 |***CmNAC1* promoter sequence of pumpkin. The 1069 bp upstream from the start codon of *CmNAC1* was cloned from pumpkin using GenomeWalker Universal Kits.

**FIGURE S6 |** Multiple alignment of NAC domain proteins from pumpkin transcriptome. Alignment was performed using DNAMAN program. Seventy six NAC domain proteins were analyzed.

**FIGURE S7 |** Phylogenetic relationship of CmNAC1 with SNACs proteins from different crops. SNAC1, SNAC3, OSNAC48 from *Oryza Sativa*, SlNAC4 from *Solanum lycopersicum*, GhNAC2 from *Gossypium hirsutum*, GmNAC2 from *Glycine max*, and AtATAF1 from *Arabidopsis thaliana.*

## Abbreviations

ABA: abscisic acid
ABRE: ABA response elements
DAPI: 4′,6-diamidino-2-phenylindole
DIC: differential interference contrast images
GA: gibberellin
NARD: NAC repression domain
SNACs: stress-responsive NAC transcription factors
