# Supplementary figures and images for: Ectopic Expression of Pumpkin NAC Transcription Factor CmNAC1 Improves Multiple Abiotic Stress Tolerance in Arabidopsis
Source: Front Plant Sci. 2017 Nov 28;8:2052. doi: 10.3389/fpls.2017.02052 (PMC5712414; doi:10.3389/fpls.2017.02052)

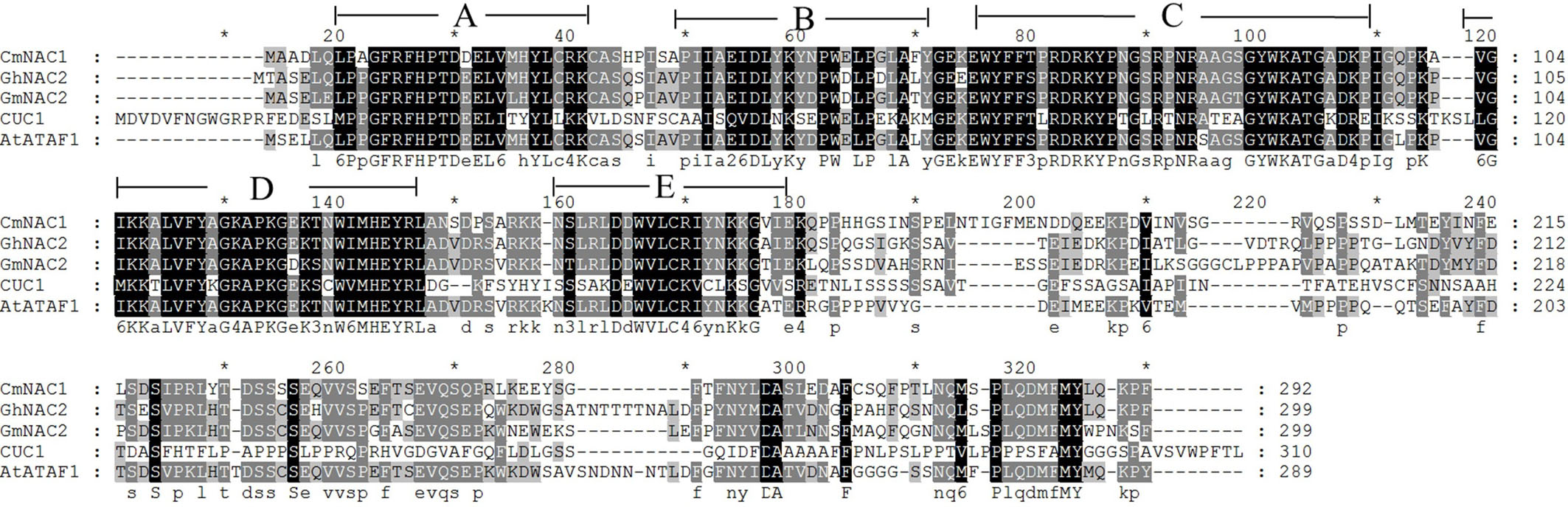

Supplement: Supplementary file 1 [file Image_1.JPEG]

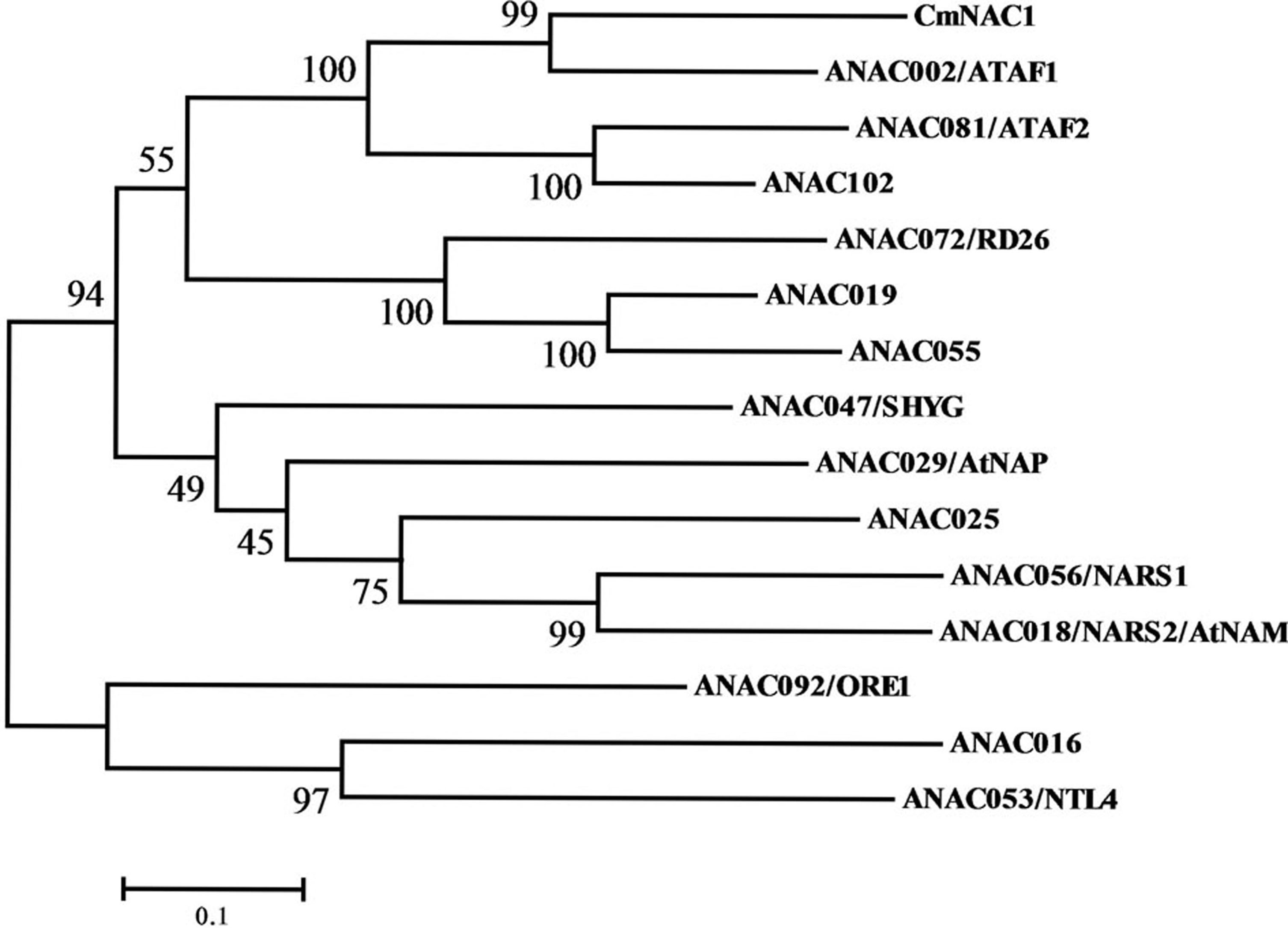

Supplement: Supplementary file 2 [file Image_2.JPEG]

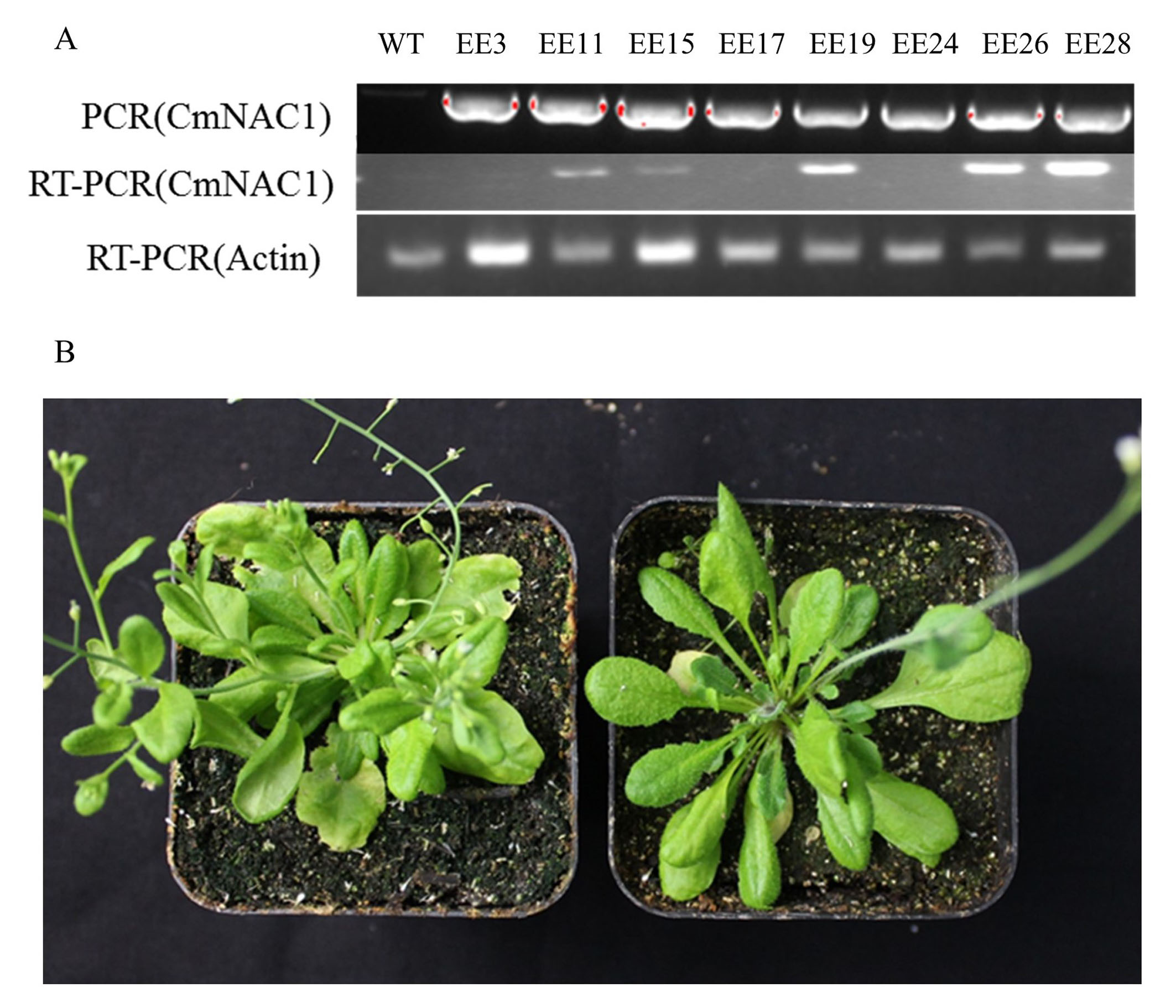

Supplement: Supplementary file 3 [file Image_3.JPEG]

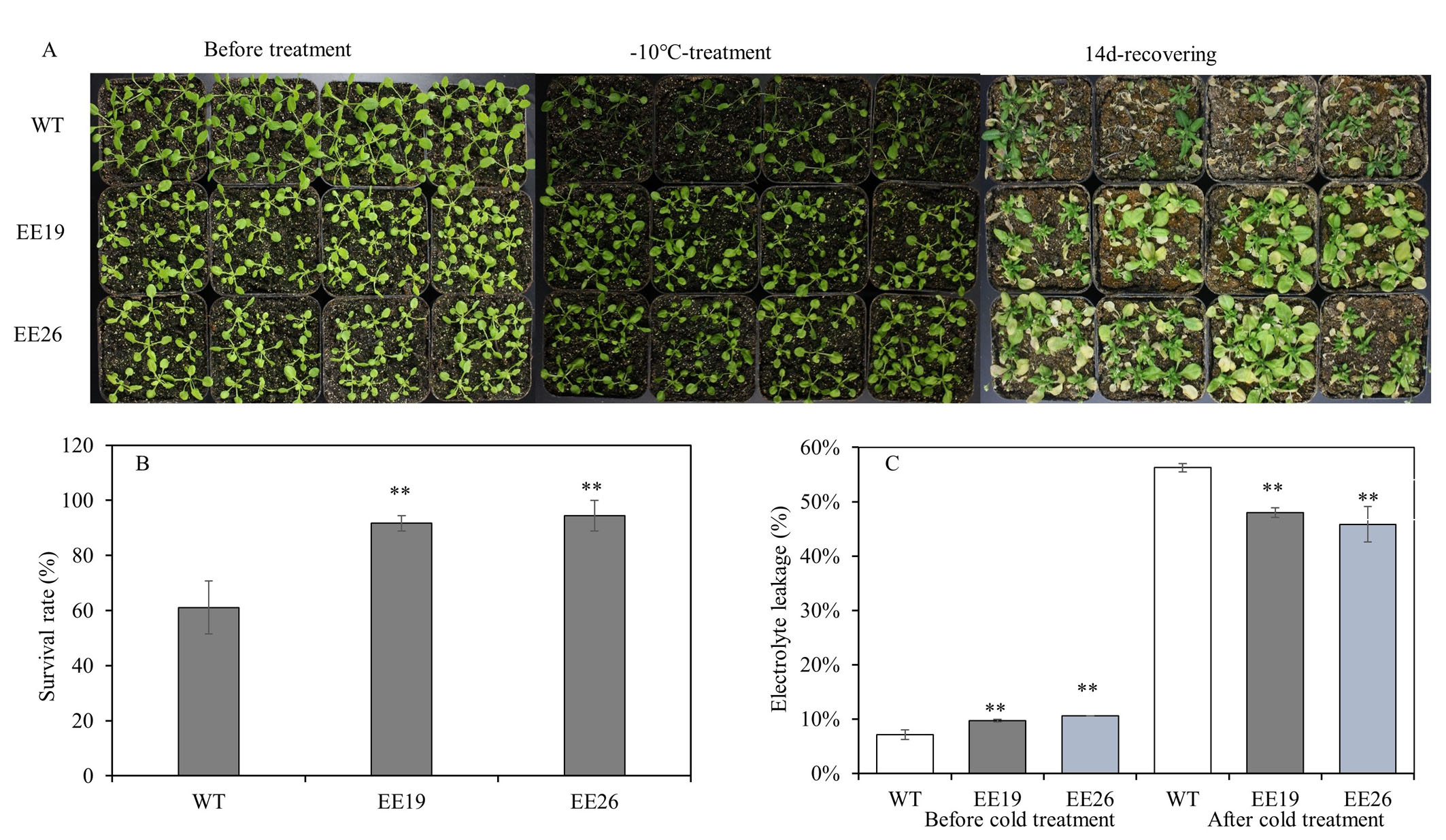

Supplement: Supplementary file 4 [file Image_4.JPEG]

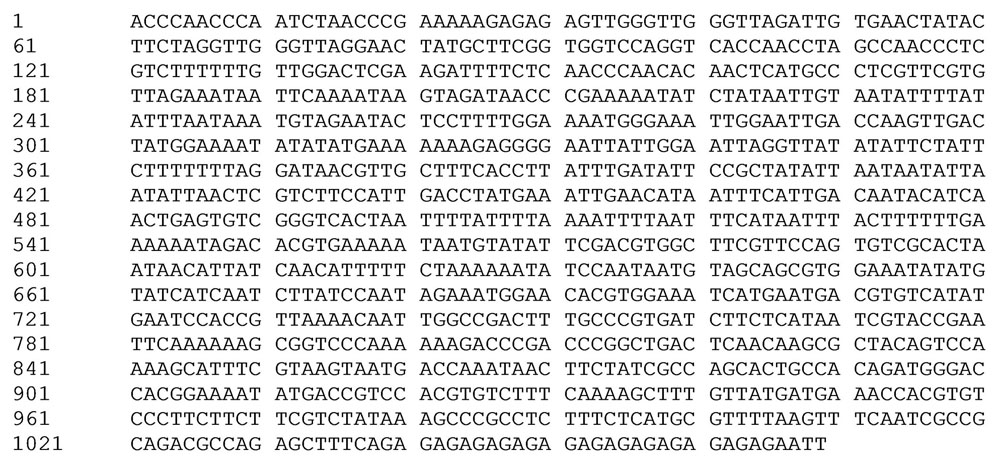

Supplement: Supplementary file 5 [file Image_5.JPEG]

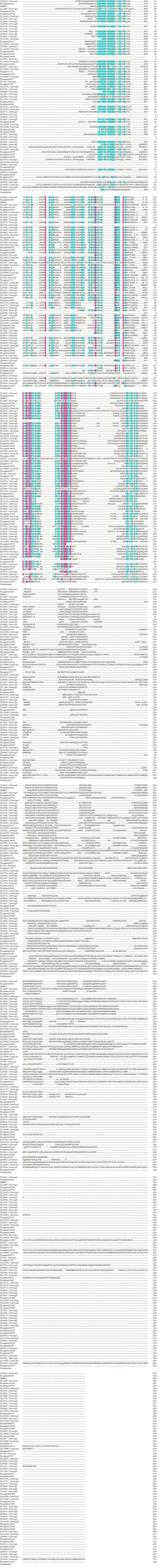

Supplement: Supplementary file 6 [file Image_6.JPEG]

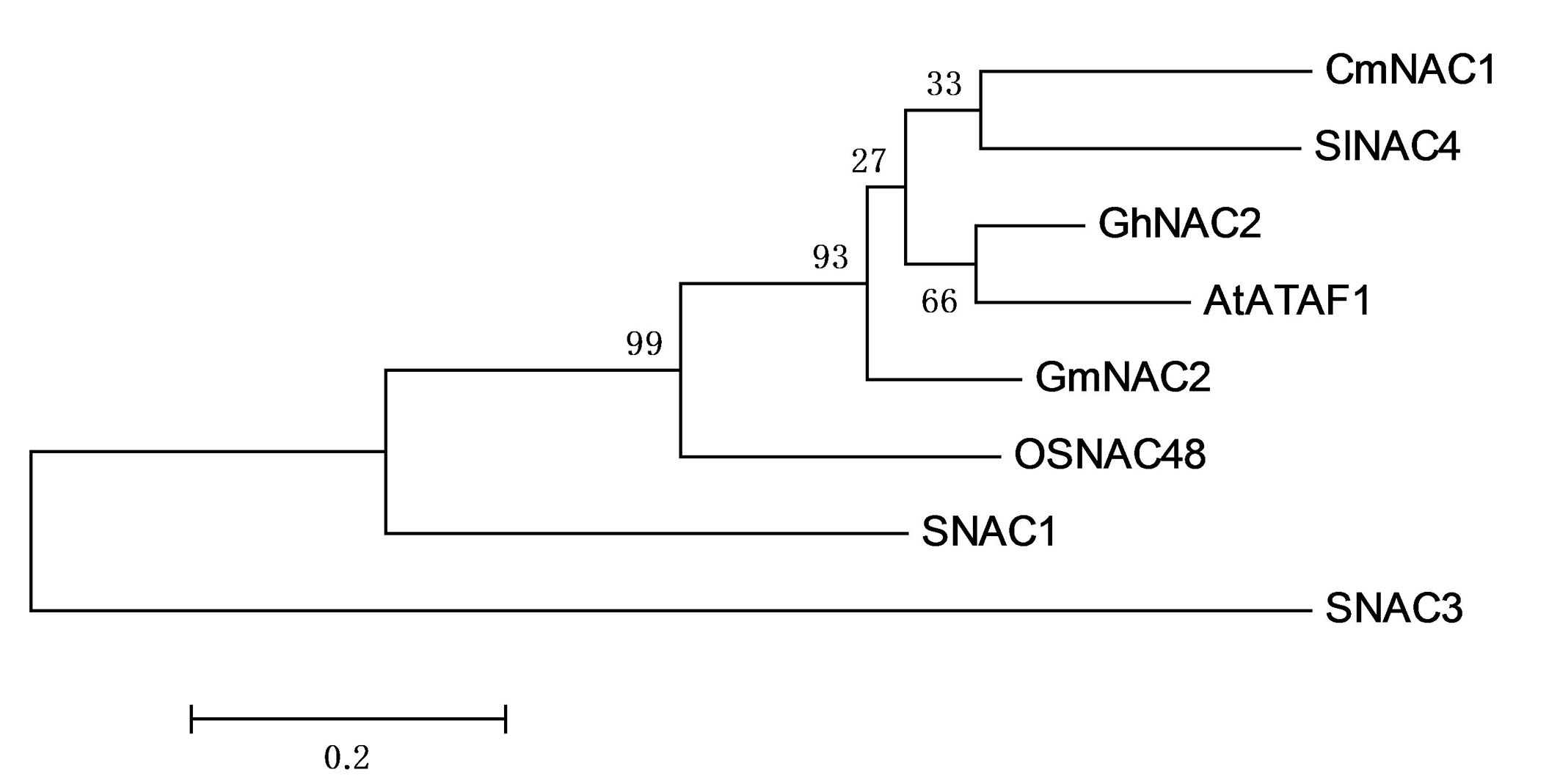

Supplement: Supplementary file 7 [file Image_7.JPEG]
