# Supplementary material for: Ectopic Expression of Pumpkin NAC Transcription Factor CmNAC1 Improves Multiple Abiotic Stress Tolerance in Arabidopsis
Source: Front Plant Sci. 2017 Nov 28;8:2052. doi: 10.3389/fpls.2017.02052 (PMC5712414; doi:10.3389/fpls.2017.02052)
Supplement: Supplementary file 8 [file Table_1.DOCX]

**Supplementary Table 1.** ABREs identified in the promoter region of *AtATAF1* (*Arabidopsis thaliana*) and *CsNAC1* (*Cucumis sativus*).

| Site Name | Organism | Position | Strand | Matrix score. |  | sequence |
| --- | --- | --- | --- | --- | --- | --- |
| ABRE | Cucumber | 142,404 | + | 6 |  | CACGTG |
| ABRE | Cucumber | 106,108,140,  181,183 | - | 6 |  | CACGTG |
| ABRE | *Arabidopsis thaliana* | 191,193,288 | - | 6 |  | CACGTG |
| ABRE | *Arabidopsis thaliana* | 232,260 | + | 6 |  | CACGTG |
