# Supplementary material for: Ectopic Expression of Pumpkin NAC Transcription Factor CmNAC1 Improves Multiple Abiotic Stress Tolerance in Arabidopsis
Source: Front Plant Sci. 2017 Nov 28;8:2052. doi: 10.3389/fpls.2017.02052 (PMC5712414; doi:10.3389/fpls.2017.02052)
Supplement: Supplementary file 9 [file Table_2.DOCX]

**Supplementary Table 2.** List of Primer sequences

| Gene | Forward primer | Rerverse primer | |
| --- | --- | --- | --- |
| For Transactivation assay | | | |
| pGBDKT7-CmNAC1-FL | AGGCCGAATTCCCGGGGATCCGTATGGCCGCCGATTTACAGTTGC | | CCGCTGCAGGTCGACGGATCCTCAAAATGGCTTCTGAAGGTACATG |
| pGBDKT7-CmNAC1-N | AGGCCGAATTCCCGGGGATCCGTATGGCCGCCGATTTACAGTTGC | | CCGCTGCAGGTCGACGGATCCCACCCAATCGTCTAACCTAAGAC |
| pGBDKT7-CmNAC1-C | AGGCCGAATTCCCGGGGATCCGTCTCTGCCGCATATACAACAAAAAAGG | | CCGCTGCAGGTCGACGGATCCTCAAAATGGCTTCTGAAGGTACATG |
| For Subcellular localization | | | |
| CmNAC1-NGFP | ATTACGCCGAGGTCATGGCCGCCGATTTACAGTT | | TAGGGAAGAGGTCAAAATGGCTTCTGAAGGTAC |
| For gene amplification | | | |
| CmNAC1-Pro | CAACGGCCCATCTGAGAACC | | AATTCTCTCTCTCTCTCTCTCTCTC |
| CmNAC1-gate8 | CATTTGGAGAGGACACGCTCGAGATGGCCGCCGATTTACAGTTGC | | TCTCATTAAAGCAGGACTCTAGATCAAAATGGCTTCTGAAGGTACATG |
| For RT-PCR | | | |
| CmNAC1-RT-PCR | GGGATTAAGAAGGCGTTGGT | | GAACTCCGACGACATCACCT |
| CmNAC1-RT-Qpcr | GAAGCCGGATGTGCTCAACG | | GAAGCCGGATGTGCTCAACG |
| CmCAC-RT-qPCR | GGACAAACAGAACCAACCATGA | | GGTTTCCTTTCCGTCACTGTAGA |
| CmEF-1α | GCTTGGGTGCTCGACAAACT | | TCCACAGAGCAATGTCAATGG |
| AtNECD3 | AGCTCCTTACCTATGGCCAG | | CGCTCTCTGGAACAAATTCATC |
| AtActin2 | CTTGCACCAAGCAGCATGAA | | CCGATCCAGACACTGTACTTCCTT |
|  |  | |  |
